# AlignerBoost: A Generalized Software Toolkit for Boosting Next-Gen Sequencing Mapping Accuracy Using a Bayesian-Based Mapping Quality Framework

**DOI:** 10.1371/journal.pcbi.1005096

**Published:** 2016-10-05

**Authors:** Qi Zheng, Elizabeth A. Grice

**Affiliations:** Department of Dermatology, Perelman School of Medicine, University of Pennsylvania, Philadelphia, Pennsylvania, United States of America; Universite de Montreal, CANADA

## Abstract

Accurate mapping of next-generation sequencing (NGS) reads to reference genomes is crucial for almost all NGS applications and downstream analyses. Various repetitive elements in human and other higher eukaryotic genomes contribute in large part to ambiguously (non-uniquely) mapped reads. Most available NGS aligners attempt to address this by either removing all non-uniquely mapping reads, or reporting one random or "best" hit based on simple heuristics. Accurate estimation of the mapping quality of NGS reads is therefore critical albeit completely lacking at present. Here we developed a generalized software toolkit "AlignerBoost", which utilizes a Bayesian-based framework to accurately estimate mapping quality of ambiguously mapped NGS reads. We tested AlignerBoost with both simulated and real DNA-seq and RNA-seq datasets at various thresholds. In most cases, but especially for reads falling within repetitive regions, AlignerBoost dramatically increases the mapping precision of modern NGS aligners without significantly compromising the sensitivity even without mapping quality filters. When using higher mapping quality cutoffs, AlignerBoost achieves a much lower false mapping rate while exhibiting comparable or higher sensitivity compared to the aligner default modes, therefore significantly boosting the detection power of NGS aligners even using extreme thresholds. AlignerBoost is also SNP-aware, and higher quality alignments can be achieved if provided with known SNPs. AlignerBoost’s algorithm is computationally efficient, and can process one million alignments within 30 seconds on a typical desktop computer. AlignerBoost is implemented as a uniform Java application and is freely available at https://github.com/Grice-Lab/AlignerBoost.

“This is a *PLOS Computational Biology* Software paper.”

## Introduction

Numerous genome-scale experimental applications are now possible due to the advent of high throughput, low cost next-generation sequencing (NGS) platforms, including genome sequencing/re-sequencing, gene expression profiling, mRNA splicing prediction/characterization, SNP identification and genotyping, and disease-associated variant identification. Accurate mapping of NGS reads to reference genomes is critical to all of these applications. Many public or commercial NGS read mapping programs (“aligners”) are available, most of which utilize a "seed-search" first strategy to allow ultra-fast processing. The most commonly used algorithms for seed-search are Hash-index (e.g. MAQ, GSNAP, SRMapper, mrsFAST-Ultra, SeqAlto [1–5]), "Burrows-Wheeler Transform" (e.g. Bowtie/Bowtie2, BWA, SOAP2 [6–10]), un-compressed tries (e.g. STAR [11]), or a mixture of the above (e.g. YOABS [12]). These seed-search algorithms usually use relatively small segments of the reads ("seeds") to initiate mapping, due to large RAM requirements to build the index. They then attempt to extend the mapping either by naive comparison or local Smith-Waterman alignment algorithm [1–4, 6–12]. Theoretically, the use of relatively small "seeds" should provide enough uniqueness (or mapping precision) even for very large genomes. In reality however, most genomes, especially those of higher eukaryotes, are enriched with various large and highly similar repetitive elements, such as pseudogenes, paralog gene families, transposable elements, tandem repeats and sequences encoding repeat RNAs. This often leads to multiple hits in the "seed-search" stage and subsequent ambiguously mapped reads for most real NGS datasets, even those designed to target exome regions. In fact, certain NGS aligners treat seeds in highly repetitive regions as “high complexity” and ignore them by default. However, some repetitive elements in the human genome can have an overwhelming high copy number. For example, some human pseudogene classes may have more than 500 copies of over 3,000 bp; a few human SINE retrotransposon families may have over 100,000 copies of about 300 bp. In these repetitive regions, a “low complexity” seed might not even exist, leading to biased mapping in favor of these regions and subsequent false mapping.

Incorrect mapping of NGS reads may cause many problems in downstream data analyses, including biased genome/transcriptome profiling, false prediction of novel genes/transcripts, false SNP prediction, or even identification of false disease variations [13, 14]. Most current NGS aligners attempt to address this problem by either removing or suppressing all multiple-mapped reads [6], reporting a random hit [7], or reporting a "best" hit [2, 8, 9, 12]. However, all currently available “best hit” methods are based on heuristic instead of strict statistical inference, such as number of seed mismatches or Smith-Waterman alignment scores, whose effect haven't been proven for finding the correct mapping loci.

Here we present AlignerBoost, a generalized software toolkit suitable for most NGS studies requiring alignment to a reference genome. AlignerBoost significantly increases mapping precision of NGS aligners, without significantly decreasing the mapping sensitivity when only considering the best hits, especially for reads generated from repeat regions. AlignerBoost achieves this by first tuning NGS aligners to report all potential alignments, then utilizes a Bayesian-based framework to accurately estimate the mapping quality of ambiguously mapped reads.

## Results

We tested AlignerBoost with both simulated and real datasets under various combinations of experimental strategies. Since it is very difficult to determine whether a read from a real dataset is mapped correctly, we first generated synthetic NGS datasets under complex sequencing error models (see below). It is noteworthy that we didn’t choose published software for this purpose, such as SlnC [15], XS [16], GemSIM [17], or ART [18], because to our knowledge, they do not support generating simulated reads in designated regions of the genome as our procedures do. For all datasets, we tested the overall mapping precision (positive prediction value, or PPV = TP/(TP + FP) and sensitivity (true positive rate, or TPR = TP/(TP + FN)) for AlignerBoost with various NGS aligners, and compared the AlignerBoost filtered results to the default "best" outputs (S1 Table). To calculate the mapping precision and sensitivity, a “correct-mapping” is defined as aligned boundaries that are within +/- 20% of the true locus relative to the alignment length. The exact definition of a true locus is explained for simulated and real datasets separately below.

### Simulated DNA-seq datasets

We generated simulated DNA-seq datasets with a complex sequencing error model by the following procedures: (1) Random genomic regions with particular genetic features are drawn from uniform distributed locations and truncated Gaussian size-distributions; (2) Genomic single-end (SE) or paired-end (PE) reads with given size are extracted from these regions; (3) Simulated base qualities (sequencing errors) are randomly drawn from Gaussian distributions with fixed mean qualities at seed regions and progressively decreasing means at subsequent bases both with fixed standard-deviations, for forward and reverse reads independently; (4) Original reads are then subjected to a mutation process for substitutions, insertions, and deletions according to designated base qualities. The resulting simulated NGS datasets have many common features of datasets produced by modern NGS sequencing platforms, such as variable base qualities both for different reads and different positions (S1 Fig). To mimic different experimental designs, we generated four simulated DNA-seq datasets of four different genetic features, namely total genome (Genome), RefSeq gene exons (RefExome), VegaPseudogenes (Pseudogene), and Repeat-Masker annotated Transposable Elements (RMSK). Detailed parameters for generating these simulated NGS datasets can be found in S2 Table.

**Fig 1:**
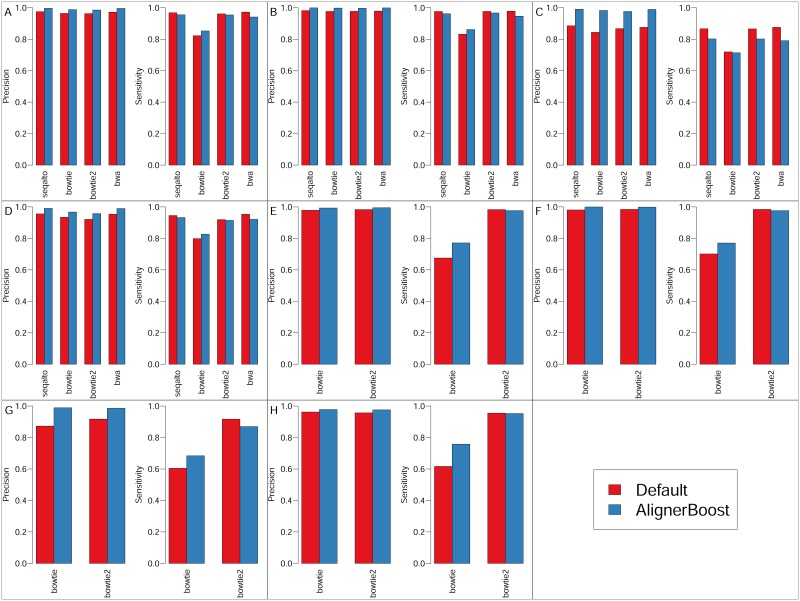
Mapping sensitivity and precision of simulated DNA-seq datasets by picking “best” hits using AlignerBoost filtering procedures (AlignerBoost) or the aligner’s default best mode (Default). A-D: Single-end (SE) mapping; E-H: Paired-end (PE) mapping; A/E, B/F, C/G, D/H: SE/PE results for Genome, RefExome, Pseudogene and RMSK simulated datasets, respectively.

We mapped all simulated datasets with or without using AlignerBoost coupled with 4 NGS aligners, namely SeqAlto [5], Bowtie [6], Bowtie2 [7] and BWA-MEM (abbreviated to “BWA” hereafter) [8, 9], then compared the “best” alignments (with highest mapQ values) between AlignerBoost filtered or the program’s default outputs. In general, the filtered results achieved much higher mapping precision (most times >97%) without significantly losing sensitivity (sometimes even with increased sensitivity) compared to the "default" results (Fig 1, S3 and S4 Tables), regardless of which type of aligner was used. This is especially true for the repeat-rich datasets, i.e. pseudogenes and RMSK, where the precision gain can be very profound (up to ~15%). As expected, for PE-reads the default method already yielded reasonably good mapping precision especially for genome and RefExome datasets, yet AlignerBoost achieved even higher precision (mostly >98%) and with no sensitivity losses (Fig 1E–1H and S4 Table). Therefore, AlignerBoost is suitable for most, if not all, NGS experimental designs, especially for those with many repeat-oriented sequences and relatively short read length. It is of note that some aligners, such as SeqAlto and BWA, don’t support multiple mapping under PE mode, and were therefore not tested under these scenarios.

**Fig 2:**
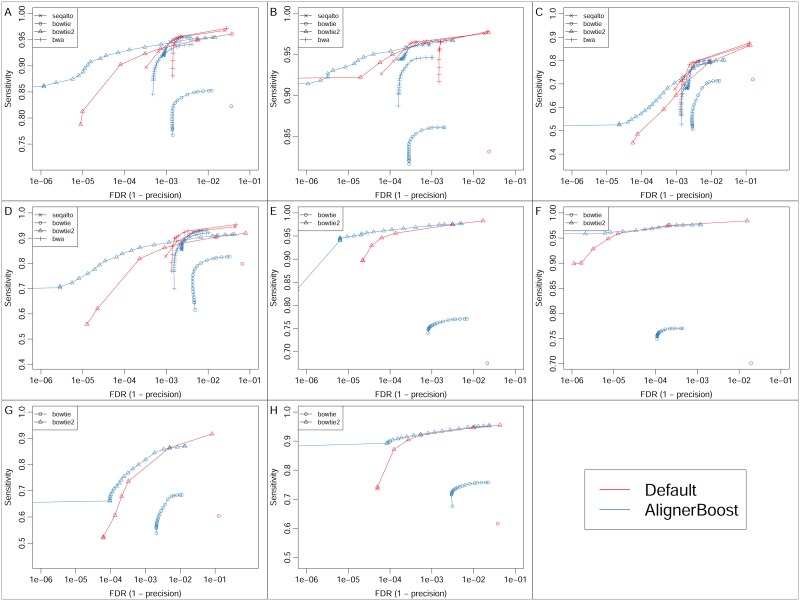
The mapping sensitivity vs. False Discovery Rate (FDR) curves under different mapping quality (mapQ) cutoffs for the simulated DNA-seq datasets. The mapQ varies from 0, 3, 6, 10, 13, 20, then in increments of 10 up to the maximum allowed values of the indicated aligner. “Default” indicates aligners’ default best hits; “AlignerBoost” indicates best hits via AlignerBoost mapping and filtering procedures. A-D: Single-end (SE) mapping; E-H: Paired-end (PE) mapping; A/E: Genome, B/F: RefExome, C/G: Pseudogene, D/H: RMSK.

It is not uncommon that extremely high precision alignment is required, such as when calling disease-related genetic variations. Since most modern NGS aligners report variable empirical mapQ values, we sought to test the read mapping performance of AlignerBoost (regarding the sensitivity and FDR (1- precision)) by applying a minimum mapQ cutoff at different thresholds (Fig 2). Strikingly, AlignerBoost achieves up-to one order of magnitude lower FDR rate toward the very strict end of the mapQ cutoff threshold, while maintaining an equivalent or even higher sensitivity, for all 4 simulated datasets (Fig 2A–2D). Similar mapping performance improvements were observed for the PE datasets, where we only tested those aligners supporting multiple mapped reads under PE mode (Fig 2E–2H). The simultaneous gain of precision and sensitivity is very profound for aligners known to be able to carry out near-exhaustive searches (e.g. Bowtie2 and BWA), suggesting it is a good combination to use AlignerBoost with highly sensitive NGS aligners.

### Simulated RNA-seq datasets

Another widely used NGS application is RNA-seq, which has added complexity regarding read mapping, including exon/intron junction handling and post-transcription modifications, such as polyadenylation and editing. These complexities can lead to improper partial alignments between the non-genomic parts of the reads to the genome, especially for those aligners that do not implement the Smith-Waterman local alignment algorithm. To test this, we generated simulated RNA-seq datasets (both SE and PE) from RefSeq mRNAs (refGenes) using a similar procedure as for the DNA-seq datasets, with the exception that random regions were drawn directly from spliced RefSeq mRNAs instead of the genome (S2 Table). We then did similar comparisons as above but with additional NGS aligners, including two dedicated RNA-seq aligners, Tophat2 and STAR, which can handle splicing-junction alignments [11, 19]. We also enabled the "1DP" feature (see Methods) for aligners without local-alignment algorithm support for the reasons explained above.

**Fig 3:**
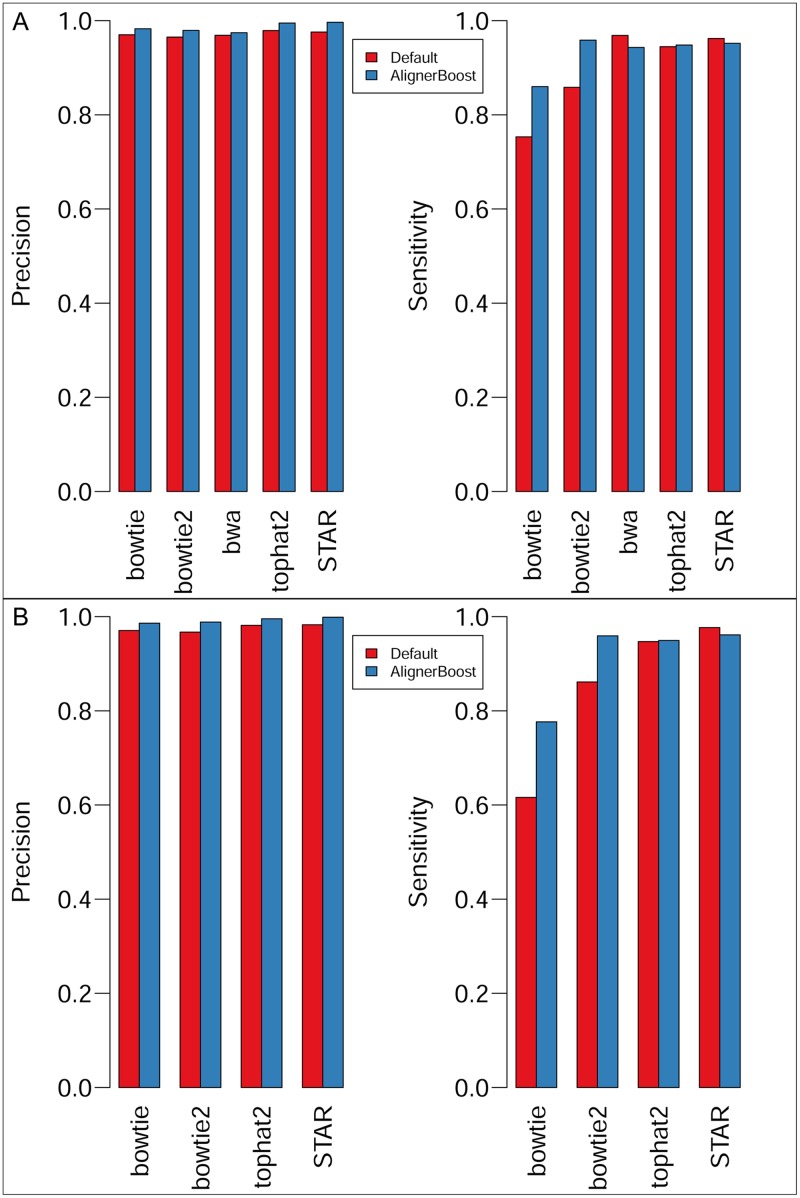
Mapping sensitivity and precision of simulated RNA-seq datasets by picking “best” hits using AlignerBoost filtering procedures (AlignerBoost) or the aligner’s default best mode (Default). A: Single-end (SE) mapping; B: Paired-end (PE) mapping.

Similar to the DNA-seq dataset results, AlignerBoost achieves significantly higher precision compared to the default results for all aligners tested and for both SE and PE reads (Fig 3, S5 and S6 Tables). Surprisingly, it often increases the mapping sensitivity as well, especially for DNA-seq aligners (Fig 3), suggesting that the default mode of these aligners for RNA-seq reads are not optimal when compared to the AlignerBoost fine-tuned options.

**Fig 4:**
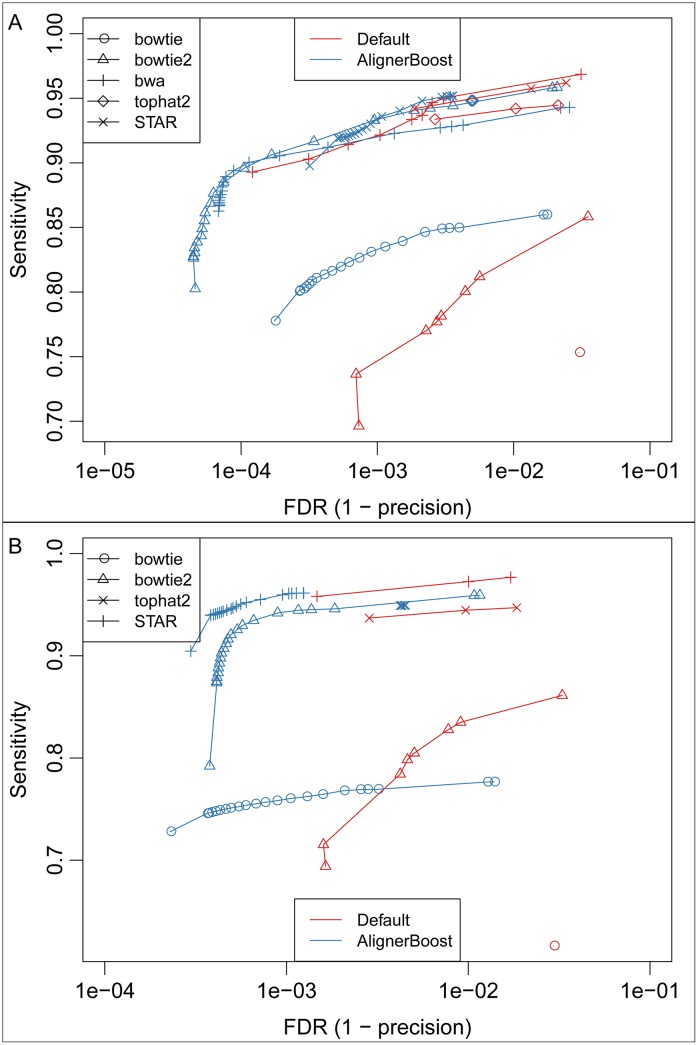
The mapping sensitivity vs. False Discovery Rate (FDR) curves under different mapping quality (mapQ) cutoffs for the simulated RNA-seq datasets. The mapQ varies from 0, 3, 6, 10, 13, 20, then in increments of 10 up to the maximum allowed values of the indicated aligner. “Default” indicates aligners’ default best hits; “AlignerBoost” indicates best hits via AlignerBoost mapping and filtering procedures. A: Single-end (SE) mapping; B: Paired-end (PE) mapping.

We also tested AlignerBoost performance under different mapQ cut-offs for the RNA-seq dataset (Fig 4). As expected, AlignerBoost achieves 1–2 orders of magnitude smaller FDR without significantly losing (sometimes even strongly gaining) mapping sensitivity, which is even true for the PE-reads (Fig 4B). Notably by using AlignerBoost, the overall mapping quality (regarding sensitivity and FDR) of the DNA-seq aligners is comparable to the dedicated RNA-seq aligners, suggesting that it is technically practical to use DNA-seq aligners along with AlignerBoost for RNA-seq mapping purposes, especially in cases that reliable annotation of splicing isoforms is not available, and the RNA-seq experiments serve mainly as a Next-Gen approach to determine differential gene expression.

### Real datasets

Real experimental NGS datasets are much more complicated than simulated datasets; besides sequencing error, incorrect mapping could be caused by SNP, CNV, chromosome rearrangement, RNA modification/editing, etc. It is very difficult to judge the effects of these elements on mapping due to a lack of "gold-standard" datasets. As a first effort, we selected public exome-sequencing (exome-seq) datasets from NCBI SRA, which utilized 4 widely-used commercial target-enrichment kits, namely Agilent SureSelect v4.0, Agilent Haloplex V3, NimbleGen SeqCap EZ v3.0, and Illumina TruSeq Exome (abbreviated hereafter to SureSelect, Haloplex, SeqCap EZ, TruSeq, respectively), all of which are paired-end datasets. These kits are designed to selectively purify pre-defined coding genomic regions (exome) that cover ~51.2 Mb, ~105.8 Mb, ~64.2 Mb and ~62.1 Mb of the human genome, respectively. They therefore provide a decent proxy for determining "correct mapping" of NGS reads. To be specific, a "correctly mapped" read is defined when its mapped locus overlaps to any of the designed capture regions.

**Fig 5:**
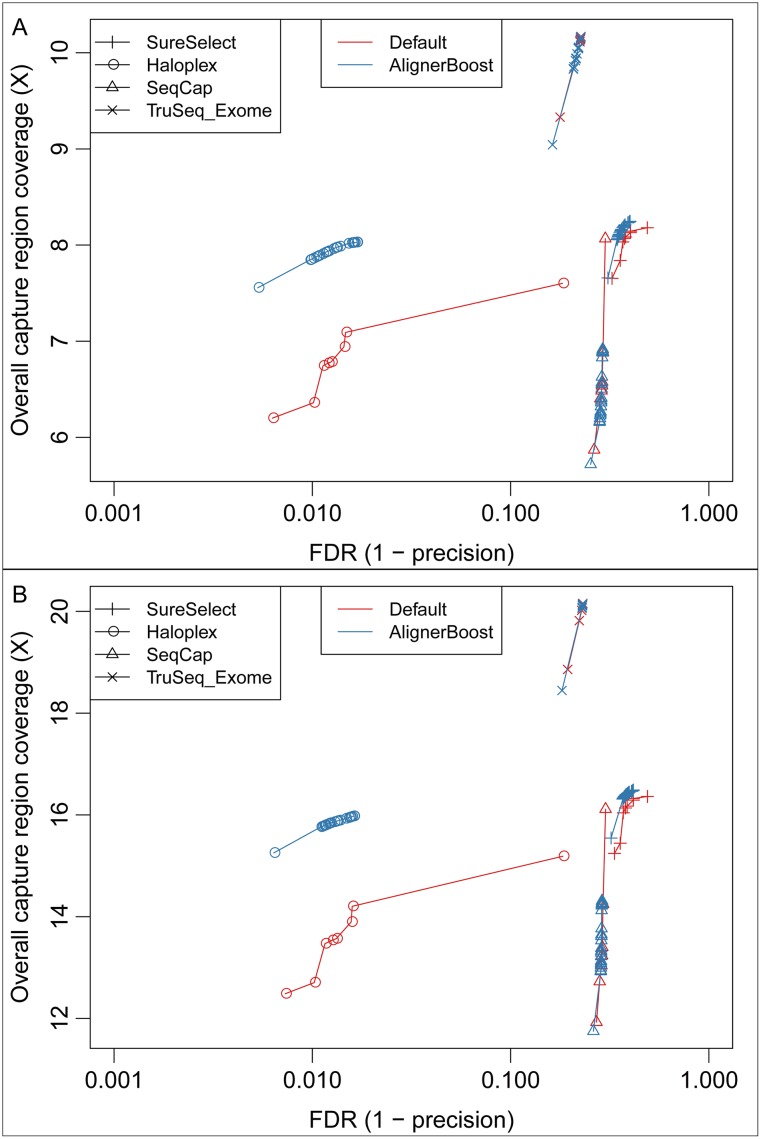
The estimated mapping sensitivity vs. False Discovery Rate (FDR) curves under different mapping quality (mapQ) cutoffs for the real exome-seq datasets. All mappings were performed using Bowtie2. Mapping sensitivity is approximated by the read depth in capture regions. The mapQ varies from 0, 3, 6, 10, 13, 20, then in increments of 10 up to the maximum allowed values of the indicated aligner. “Default” indicates aligners’ default best hits; “AlignerBoost” indicates best hits via AlignerBoost mapping and filtering procedures. A: Single-end (SE) mapping; B: Paired-end (PE) mapping.

We randomly down-sampled all 4 exome-seq datasets to 10 million reads and generated pseudo SE-datasets by dropping all reverse reads. These real datasets exhibit a very similar quality pattern as our simulated dataset (S2 Fig). We tested them similarly as described for the simulated datasets; note that all mappings were performed using the Bowtie2 aligner due to its superior performance in conjunction with AlignerBoost as shown in the simulated results. Since the exact true loci of these reads are unknown, we evaluated the total coverage depth of designed capture regions instead of mapping sensitivity, and the mapping precision should be treated as estimation. In general the result is very similar to the simulated datasets: if just considering the best hits without any mapQ restriction, AlignerBoost strongly increases the precision without significant loss or even gain of mapping sensitivity, while toward the extreme mapQ cutoffs, AlignerBoost achieves a noticeably lower FDR rate with increased sensitivity (Fig 5). A very similar overall mapping performance improvement is observed for both SE and PE reads (Fig 5A and 5B). This fact holds true when we switched to the BWA (BWA-MEM) aligner (S3 Fig), indicating the observed performance improvement is not specific to a particular aligner. Notably, even with AlignerBoost filtering, the highest achieved precision was usually <90% (FDR > 0.1) except for the Agilent Haloplex platform; this is potentially caused by non-specific binding between synthesized probes and target DNAs (i.e. binding to pseudogenes/repeats), and exaggerated by the possibility that flanking sequences of target regions were pulled down during capture but after random fragmentation did not overlap capture regions. In fact, the estimated mapping precisions are in agreement with previous studies [20], though our results have consistently higher precision.

As discussed above, genetic variation could affect the mapping quality of NGS reads. To test this, we provided the Hap-Map phase 3 (HapMap3) or 1000genomes (1000G) SNPs to AlignerBoost to re-analyze the exome-seq datasets. Not surprisingly, there were only subtle gains of mapping precision when providing AlignerBoost with known SNPs (S7 Table), since only a very small proportion (2~3%) of reads contained any known SNPs. Interestingly, the overall coverage depth of target regions was also slightly increased when known SNPs were incorporated, giving AlignerBoost an additional advantage in detecting and confirming genetic variants near highly variable regions in the genomes.

**Fig 6:**
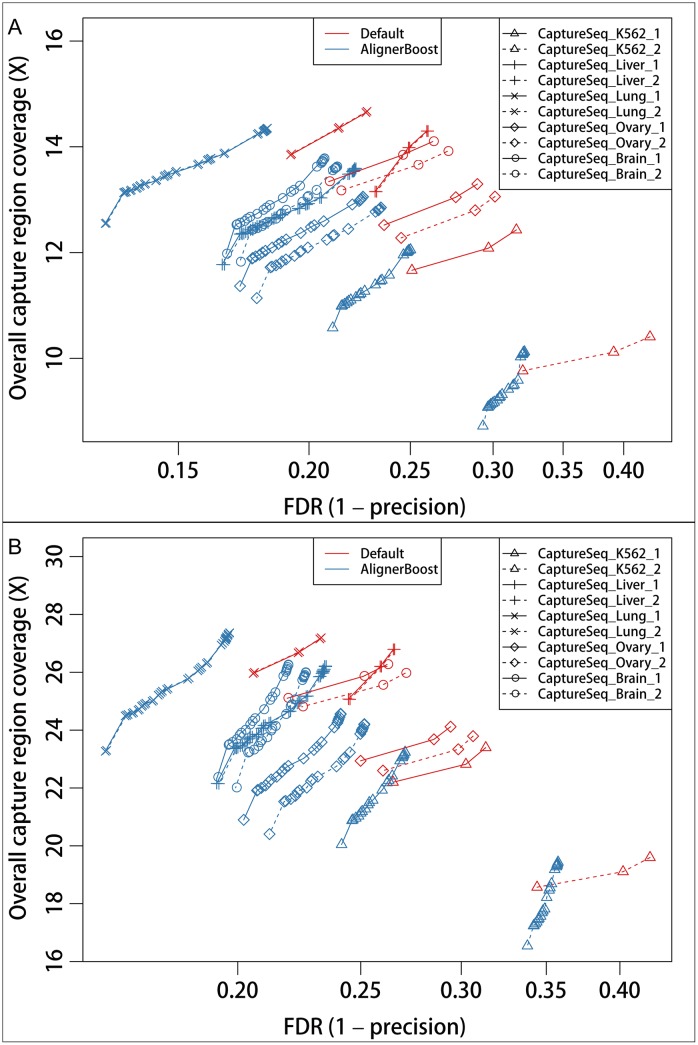
The estimated mapping sensitivity vs. False Discovery Rate (FDR) curves under different mapping quality (mapQ) cutoffs for the real capture-seq datasets. All mappings were performed using STAR. Mapping sensitivity is approximated by the read depth in capture regions. The mapQ varies from 0, 3, 6, 10, 13, 20, then in increments of 10 up to the maximum allowed values of the indicated aligner. “Default” indicates aligners’ default best hits; “AlignerBoost” indicates best hits via AlignerBoost mapping and filtering procedures. A: Single-end (SE) mapping; B: Paired-end (PE) mapping. Replicate samples have same point types but different line types.

A recent option for RNA-seq studies is to apply target enrichment similar to that of exome-seq [21, 22] experiments. This new approach, termed Capture-seq, gives us an opportunity to closely examine the effect of AlignerBoost in RNA-seq studies. We compared the AlignerBoost filtered vs. default alignments for 10 public Capture-seq libraries from human tissues and cells, and found a profound reduction of the FDR, although the overall read depth of designed capture regions moderately suffered (Fig 6A and 6B).

**Fig 7:**
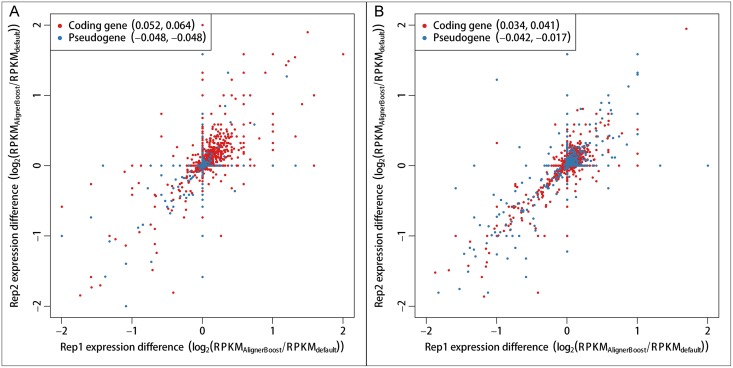
Gene expression differences between AlignerBoost filtered or “default “best alignments for two replicate Capture-seq datasets from human brain tissues. Gene expression is represented by RPKM values. Coding gene (red) and pseudogene (blue) annotations are from GENCODE project (v19). Values in parentheses show the mean gene expression changes of the two replicates. A: Single-end (SE) mapping; B: Paired-end (PE) mapping.

As discussed above, it is usually more difficult to estimate the mapping accuracy for RNA-seq data, mainly due to the potential spatial separation between capturing probes and sequencing reads by the presence of large introns. We therefore further evaluated the general effect of AlignerBoost on gene expression profiling using the Capture-seq libraries by comparing the normalized transcript expression (in Read per Kb per Million or RPKM) on a gold standard coding mRNA dataset (coding gene) as well as a pseudogene dataset (pseudogene) with or without using AlignerBoost. Not surprisingly, compared to the default results, AlignerBoost consistently increased gene expression of the coding genes, where the pseudogene expression is decreased globally (Fig 7 and S4 Fig), especially for SE mapping (all p-values < 0.01 for both replicates, paired t-tests). These results strongly indicate improved mapping accuracy of real datasets, given that gold standard coding genes are more likely to be expressed than the pseudogenes in the tissues and cells we tested.

Taken together, AlignerBoost has practical implications in improving mapping accuracy in most reference genome-based NGS projects, thereby enabling more accurate downstream analyses and better interpretation of results.

### Comparison to similar tools

**Fig 8:**
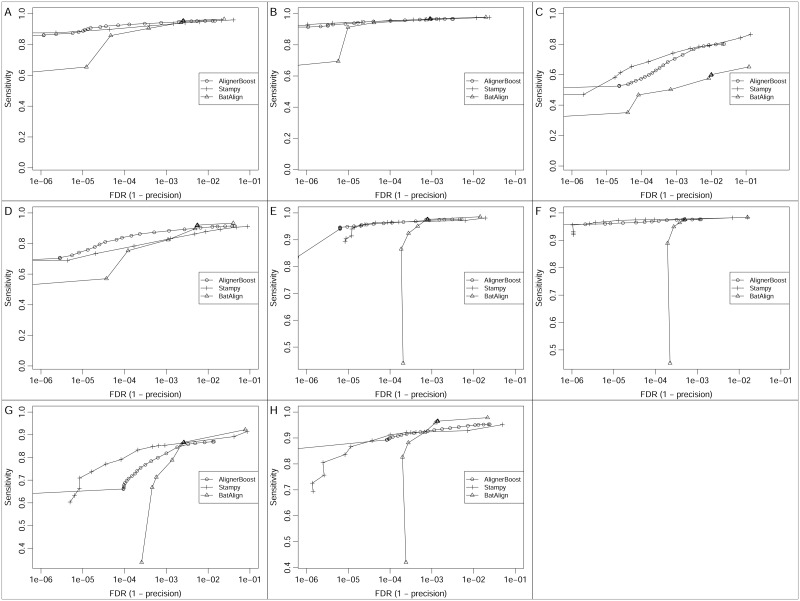
The mapping sensitivity vs. False Discovery Rate (FDR) curves under different mapping quality (mapQ) cutoffs for the simulated DNA-seq datasets using AlignerBoost and similar tools. The mapQ varies from 0, 3, 6, 10, 13, 20, then in increments of 10 up to the maximum allowed values of the indicated aligner. Different tools are labelled with different line points. AlignerBoost is used with Bowtie2 aligner. A-D: Single-end (SE) mapping; E-H: Paired-end (PE) mapping; A/E: Genome, B/F: RefExome, C/G: Pseudogene, D/H: RMSK.

AlignerBoost is designed uniquely as *ad hoc* alignment optimization software to improve NGS read mapping precision and overall performance. Although AlignerBoost is not an NGS aligner, several published NGS aligners estimate mapping quality based on similar probabilistic frameworks as AlignerBoost, such as Stampy [23] and BatAlign [24]. Stampy is a hybrid NGS aligner that first uses BWA to map reads with close representatives in the reference dataset, then maps the remaining reads using a hash based algorithm, in which large indels are treated particularly carefully. BatAlign is an incremental method for accurate read alignment, which integrates two novel strategies called “Reverse-Alignment” and “Deep-scan”. We compared the AlignerBoost filtered results (with Bowtie2 aligner) of all the simulated DNA-seq datasets to the default mapping results (running options in S1 Table) of Stampy and BatAlign in a similar way of choosing different mapQ cut-offs (Fig 8). As expected, all three tools performed similarly overall for genome and refExome datasets (Fig 8A, 8B, 8E and 8F). For RMSK datasets, AlignerBoost clearly exhibits the overall best performance (Fig 8D and 8H); for pseudogene datasets, Stampy performed best overall, while AlignerBoost reached the same level of low FDR but with relatively lower sensitivity (Fig 8C and 8G). This result suggests that for pseudogene datasets the true mapping loci are often missed by the BWT (Burrows–Wheeler transform) algorithm-based aligners tested in this study, while hash-based algorithms (such as Stampy) are more sensitive. However, a disadvantage of hash-index based NGS aligners, including Stampy, is that processing speeds are usually much slower [23].

## Discussion

Increasing throughput and decreasing costs of employing NGS platforms for various genome-wide experimental applications have made fast and accurate mapping of NGS reads to reference genomes an imperative need. Though ultra-fast speed has been achieved in many state-of-art NGS aligners, rarely have there been attempts to improve the mapping quality in terms of precision and sensitivity. Here, we developed a generalized software toolkit, AlignerBoost, which we show dramatically boosts the mapping precision for most modern NGS aligners while maintaining a similar level of sensitivity. AlignerBoost works for almost any experimental design requiring alignment to reference genomes, but has the greatest advantage for NGS libraries with a considerable proportion of repetitive reads, such as pseudogenes, transposons and paralog gene families that are usually contributing more than half of higher eukaryotic genomes. AlignerBoost supports numerous customizable mapping parameters and users can expect up to 100% mapping precision in most cases if parameters are correctly chosen. The fact that pure pseudogene or RMSK datasets can achieve up to 98% mapping precision makes it practical to interrogate these “dark matter” genomic regions with good confidence when using AlignerBoost with relatively short NGS reads. AlignerBoost is also able to greatly increase the mapping precision and sensitivity simultaneously for RNA-seq datasets regardless of whether a dedicated RNA-seq aligner is used or not, making it especially useful when mapping RNA reads to a poorly annotated genome. Furthermore, the ability to estimate the true "inserts" by "1DP" function of AlignerBoost makes it particularly promising for mapping NGS reads with non-genome fragments, which could result from untrimmed adapters/barcodes, RNA modification, exon/intron boundaries or chimeric reads. At last, we speculate that AlignerBoost will become a powerful tool for identification of disease-associated mutations and variations in the near future, when personalized SNP and variation data will be available that can be utilized by AlignerBoost to generate extremely high quality alignments.

## Methods

AlignerBoost first generates executable scripts that call external NGS aligners in multiple-mapping enabled mode. To achieve optimal sensitivity, AlignerBoost also performs optional pre-processing procedures such as quality-control (QC), adapter trimming, non-redundant tag reduction and provides sequence statistic summaries. All of these pre-processing and mapping steps are governed by tunable options, which are specified by a single user-provided configuration file, and support many major NGS aligners (S8 Table). The executable scripts generate standard SAM/BAM alignment files that contain all potential alignments (multiple-mapping enabled) for every read.

### Mapping quality of single-end reads

The core function of AlignerBoost is to estimate the mapping quality (*mapQ*) of an alignment between an NGS read and a reference genome, given all potential alignments (multiple mapping) of that read. In this application, the *mapQ* is the phred-scaled posterior probability of a mapping given all potential alignments, defined as:

$$mapQ_{i}=-10\times \text{lg}\left(1-\text{Pr}\left(map_{i}|Α\right)\right)$$ (1)

where *map*_*i*_ and *A* is the i^th^ mapping/alignment and all potential alignments for this read, respectively. AlignerBoost uses a simple Bayesian method to calculate the posterior probability by determining the likelihood of all potential alignments, as:

$$\begin{array}{lll} \text{Pr}\left(map_{i}|Α\right) & = & \frac{\text{Pr}\left(loc_{i}\right)\text{Pr}\left(align_{i}|loc_{i}\right)}{\sum_{k\in Α}\text{Pr}\left(loc_{k}\right)\text{Pr}\left(align_{k}|loc_{k}\right)}\\ & = & \frac{1}{\tilde{Z}}L_{i}\text{Pr}\left(align_{i}|loc_{i}\right) \end{array}$$ (2)

where *L*_*i*_ is the alignment length, Pr(*align*_*i*_ | *loc*_*i*_) is the alignment likelihood for this locus, and $\tilde{Z}$ is the normalization constant. Note that we use the widely-accepted prior probability of a mapping from any given locus which is proportional to its alignment length as Pr(*loc*_*i*_) = *L*_*i*_ / *L*_*G*_ where *L*_*G*_ is the effective genome size. The alignment likelihood above is calculated in log10-scale, as:

$$\begin{array}{l} \text{lgPr}\left(align_{i}|loc_{i}\right)=\sum_{j=1}^{L_{i}}\text{lgPr}\left(A_{ij}|Q_{ij}\right)\\ =\sum_{j=1}^{L_{i}}\left\{\begin{array}{ll} \text{lg}\left(1-Q2P\left(Q_{ij}\right)\right) & A_{ij}=\text{match}\\ Q_{ij}/s & A_{ij}=\text{mismatch}\\ Q_{ij}/s-\gamma_{o} & A_{ij}=\text{gap-open}\\ Q_{ij}/s-\gamma_{e} & A_{ij}=\text{gap-ext}\\ Q_{ij}/s-\gamma_{s} & A_{ij}=\text{soft-clip}\\ {\hat{Q}}_{i}/s-\gamma_{h} & A_{ij}=\text{hard-clip}\\ 0 & A_{ij}=\text{N or P} \end{array}\right\} \end{array}$$ (3)

where *A*_*ij*_ and *Q*_*ij*_ is the *j*^*th*^ aligned position and base quality score (in phred scale) of the alignment, ${\hat{Q}}_{i}$ is the estimated base quality of the (unobserved) hard-clipped bases, *γ*_*o*_, *γ*_*e*_, *γ*_*s*_, *γ*_*h*_ are the penalty scores for each alignment status, and *s* is the phredscore scaling factor (always -10 here). The *Q2P*(.) is a simple function for converting a phred-scale quality score back to the error probability as:

$$Q2P\left(q\right)=10^{-q/10}$$ (4)

Practically the ${\hat{Q}}_{i}$ was estimated using the average base quality of a small region immediately adjacent to the hard-clipped bases. Note that alignment positions with *N* (intron) or *P* (padding) status are ignored. The penalty scores used above can be considered the relative occurring chance of each status compared to a mismatch in the unit of log10 scale, and can be controlled in the configuration files.

### Mapping quality of paired-end reads

For paired-end read alignments, the likelihood of each mate of a paired alignment is calculated independently as Eq (3); however the posterior probability of a mapping pair is calculated jointly, as

$$\begin{array}{l} \text{Pr}\left(pair_{i}|Ρ\right)=\frac{\text{Pr}\left(loc_{i}\right)\text{Pr}\left(pair_{i}|loc_{i}\right)}{\sum_{k\in Ρ}\text{Pr}\left(loc_{k}\right)\text{Pr}\left(pair_{k}|loc_{k}\right)}\\ =\frac{1}{\tilde{Z}}L_{i}^{fwd}L_{i}^{rev}\text{Pr}\left(align_{i}^{fwd}|loc_{i}^{fwd}\right)\text{Pr}\left(align_{i}^{rev}|loc_{i}^{rev}\right){\text{Pr}}_{pair}\left(loc_{i}^{fwd},loc_{i}^{rev}\right) \end{array}$$ (5)

where *fwd* and *rev* stand for forward and reverse mate of the pair, respectively, and ${\text{Pr}}_{pair}\left(loc_{i}^{fwd},loc_{i}^{rev}\right)$ is the pairing probability between read mates. In practice, the pairing probability is calculated either as the Gaussian probability density of given mate distance ${\text{Pr}}_{pair}\left(loc_{i}^{fwd},loc_{i}^{rev}\right)=\varphi \left(d_{mate};\mu ,\sigma^{2}\right)$, where *μ* and *σ*^*2*^ are the estimated mean and standard deviation of the fragment size between the forward and reverse mates, if they can be reliably estimated, or as a constant if not (i.e. for RNA-seq reads with introns between read mates). It is of note that if one mate of a pair is missing (due to discordant distance, sequencing error, or other reasons), function (5) is still used but the entire missing mate is treated as if every base is "soft-masked". This treatment tends to prevent biased mapping qualities when paired-end and unpaired alignments co-exist for certain reads, so the unpaired alignments will not always be preferred over paired alignments.

### Incorporation of known variations

To accommodate known variants including single nucleotide polymorphism (SNP), indels and multiple nucleotide polymorphism (MNP), AlignerBoost can read in pre-defined variation information from standard VCF/gVCF files, and uses a slightly modified method to calculate the mapping qualities for enhanced accuracy. To be specific, the likelihood of an alignment is calculated either by function (3) or its enhanced version below:

$$\begin{array}{l} \text{lgPr}\left(align_{i}|loc_{i}\right)=\sum_{j=1}^{L_{i}}\text{lgPr}\left(A_{ij}|Q_{ij}\right)\\ =\sum_{j=1}^{L_{i}}\left\{\begin{array}{ll} \text{lg}\left(1-Q2P\left(Q_{ij}\right)\right) & A_{ij}=\text{match}\\ Q_{ij}/s & A_{ij}=\text{mismatch}\\ Q_{ij}/s-\gamma_{o} & A_{ij}=\text{gap-open}\\ Q_{ij}/s-\gamma_{e} & A_{ij}=\text{gap-ext}\\ Q_{ij}/s-\gamma_{s} & A_{ij}=\text{soft-clip}\\ \bar{Q_{i}}/s-\gamma_{h} & A_{ij}=\text{hard-clip}\\ 0 & A_{ij}=\text{N or P}\\ Q_{ij}/s-\gamma_{v} & A_{ij}=\text{known SNP}\\ Q_{ij}/s-\gamma_{g} & A_{ij}=\text{known indel}\\ Q_{ij}/s-\gamma_{b} & A_{ij}=\text{known MNP} \end{array}\right\} \end{array}$$ (6)

where the biological explanation of the penalty-scores *γ*_*v*_, *γ*_*g*_, and *γ*_*b*_ are similar as in Eq (3) for each indicated known variation types (see http://www.ncbi.nlm.nih.gov/books/NBK44447/ for their detailed definitions), and their default values can be controlled in the user-specified configuration files. For variations with allelic frequency (*AF*) available, AlignerBoost estimates these penalty-scores as

$$\gamma =-\text{lg}\left(AF_{alt}\right),$$ (7)

where *AF*_*alt*_ is the allele frequency of the observed alternate allele from the alignment. Then the largest value of the likelihood calculated by Eqs (3) or (6) calculated for each known SNP is used for subsequent *mapQ* calculation; this assumes the SNPs are independent if multiple ones are found in a same alignment, which is believed to be almost always true due to the low frequency of common SNPs and relative small NGS read size.

### Determining the actual insert range

To re-estimate the actual DNA fragment (insert) range given an alignment, a one-dimensional dynamic programming (1DP) algorithm is implemented to find the insert range that maximizes an insert score *S*; the recurrence relation of *S* is:

$$S\left(i\right)=\left\{ \begin{array}{cc} 0 & i=0\\ \text{max}\left\{\begin{array}{l} S\left(i-1\right)+A_{i}\\ 0 \end{array}\right\} & i=1,2,\ldots ,n \end{array}\right.$$ (8)

Similar to Eq (3), *A*_*i*_ is defined as the alignment score at position *i* as:

$$A_{i}=\left\{ \begin{array}{cc} a_{m} & A_{i}=\text{match}\\ a_{x} & A_{i}=\text{ mismatch}\\ -\gamma_{o} & A_{i}=\text{gap-open}\\ \;-\gamma_{e} & A_{i}=\text{ gap-ext}\\ -\gamma_{s} & A_{i}=\text{ soft-clip}\\ -\gamma_{h} & A_{i}=\text{ hard-clip}\\ 0 & A_{i}=\text{N or P} \end{array}\right.$$ (9)

where *a*_*m*_ and *a*_*x*_ have similar biological explanations as the other penalty scores described above. The mismatch and indel information is extracted from the "Cigar" elements and "MD:Z" mismatch tags from the SAM/BAM alignment records, if available.

### Other utility functions

Besides the core function of generating and filtering high quality alignments, AlignerBoost also includes many other utilities designed to fit various needs for end users, such as read QC, quick classification for SAM/BAM, BED, VCF files, and conversion from SAM/BAM files to Wig and various coverage files. The complete list of these functions can be found on the website.

### Implementations

AlignerBoost is implemented in pure Java as a unified program, similar to the latest Picard tools or GATK tools [25, 26]. For SAM/BAM file manipulations and VCF file processing, Htslib JDK from Picard tools is used and packed along with the AlignerBoost executable file. It is of note that during the 1DP process, the "Cigar" elements and "MD:Z" mismatch tags are modified to maintain the correctness of the SAM records; therefore AlignerBoost filtered alignments are ready-to-use for further analysis such as variation calls. Besides, AlignerBoost retains many mapping quality metrics such as mismatch and indel numbers and alignment likelihood as customized tags in the output BAM files. Please refer to the AlignerBoost website for a complete list of these customized tags.

### Performance

The core function of AlignerBoost is to calculate the posterior mapping probability (mapQ) of an alignment using formulas (2), (3), (5) and (6), which is in linear time complexity regarding the alignment length. The additional time and space complexity for 1DP estimation of insert range is also linear. Therefore, the overall performance of AlignerBoost is fast. In our benchmark tests, AlignerBoost can process 1 million alignments (from the total genome dataset) in 27.9 and 25.8 seconds for 100 bp SE or PE reads, respectively, on a Linux workstation using a single Intel Xeon 3.70 GHz core. These numbers are 28.2 and 29.0 seconds if we enable the 1DP function. Finally, we found that the major limitation of AlignerBoost performance is disk IO, so we didn't implement multi-threading, which often offers limited gain of processing speed at the expense of much larger memory footprint.

### Accession numbers

The NCBI SRA accession numbers for the Exome-seq datasets are SRR1609896, SRR1573550, SRR1611182 and ERR364421 for the SureSelect, Haloplex, SeqCap EZ and TruSeq enrichment kits, respectively. The SRA accession numbers for the Capture-seq datasets are SRR1576165, SRR1576167, SRR1576146, SRR1576147, SRR1576148, SRR1576149, SRR1576155, SRR1576180, SRR1576152, SRR1576177 for K562 cells, liver, lung, ovary, brain respectively, each with 2 replicates. The expression abundance (in RPKM) for all capture-seq datasets were calculated by the featureCounts program [27]. Coding gene and pseudogene annotations are downloaded from GENCODE project (v19, http://www.gencodegenes.org/), and only those overlapping with the designed capturing regions are used.

## Supporting Information

S1 Table Running options used for testing the default output of datasets. (DOCX)

S2 Table Parameters for generating simulated NGS datasets. SD: standard deviation.
(DOCX)

S3 Table Mapping sensitivity and precision of simulated DNA-seq single-end (SE) datasets by picking “best” hits with or without applying AlignerBoost procedures. AlignerBoost: AlignerBoost filtered best hits; Default: “default” best hits.
(DOCX)

S4 Table Mapping sensitivity and precision of simulated DNA-seq paired-end (PE) datasets by picking “best” hits with or without applying AlignerBoost procedures. AlignerBoost: AlignerBoost filtered best hits; Default: “default” best hits. (1) NA values are for NGS aligners that don’t support reporting all alignments under PE mode, thus AlignerBoost filtering was ineffective.
(DOCX)

S5 Table Mapping sensitivity and precision of simulated RNA-seq single-end (SE) datasets by picking “best” hits with or without applying AlignerBoost procedures. (1) DNA-seq aligner without local alignment ability, so 1DP function of AlingerBoost was enabled; (2) DNA-seq aligners; (3) RNA-seq aligners.
(DOCX)

S6 Table Mapping sensitivity and precision of simulated RNA-seq paired-end (PE) datasets by picking “best” hits with or without applying AlignerBoost procedures. (1) DNA-seq aligner without local alignment ability, so 1DP function of AlingerBoost was enabled; (2) DNA-seq aligners; (3) RNA-seq aligners; (4) NA values are for NGS aligners that don’t support reporting all alignments under PE mode, thus AlignerBoost filtering was ineffective.
(DOCX)

S7 Table Comparison of mapping precision and sensitivity of real exome-seq datasets by incorporating known SNP information for AlignerBoost. Both HapMap phase3 (HapMap3) and 1000genomes (1000G) datasets were downloaded from GATK FTP bundles. All statistics are based on best hits without mapQ cutoff.
(DOCX)

S8 Table NGS aligners currently supported by AlignerBoost. Note that AlignerBoost supports filtering customized SAM/BAM alignment files produced by ANY NGS aligners. However, for the aligners listed below, AlignerBoost supports automatic generation of executable scripts with fine tuned options aiming to boost both the precision and sensitivity of the alignments. BWT: Burrows–Wheeler transform algorithm.
(DOCX)

S1 Fig Distributions of simulated read qualities of the DNA-seq reads using the "Genome" dataset as an example. Y-axes indicate the read quality scores in Phred-scale; X-axes indicate the base-pair positions with positive and negative values representing forward and reverse read positions, respectively; Center solid lines: mean values of the quality scores at corresponding positions; shaded areas between dashed lines: mean ± SD (standard deviation) of the quality scores at corresponding positions; all quality scores were drawn from truncated Gaussian distributions. A: Quality score distributions for the simulated SE-dataset; B: Quality score distributions for the simulated PE-dataset.
(JPG)

S2 Fig Distributions of simulated read qualities using the "SureSelect" dataset as an example (SRA accession SRR1609896). Y-axes indicate the read quality scores in Phred-scale; X-axes indicate the base-pair positions with positive and negative values representing forward and reverse read positions, respectively; Center solid lines: mean values of the quality scores at corresponding positions; shaded areas between dashed lines: mean ± SD (standard deviation) of the quality scores at corresponding positions.
(JPG)

S3 Fig The mapping sensitivity vs. False Discovery Rate (FDR) curves under different mapping quality (mapQ) cutoffs for the real exome-seq single-end (SE) dataset. All mappings were performed using BWA-MEM (BWA). The mapQ varies from 0, 3, 6, 10, 13, 20, then in increments of 10 up to the maximum allowed values of the indicated aligner. “Default” indicates aligners’ default best hits; “AlignerBoost” indicates best hits via AlignerBoost mapping and filtering procedures.
(JPG)

S4 Fig Gene expression differences between AlignerBoost filtered or “default “best alignments for replicated capture-seq datasets from human cells and tissues. Gene expression represented by RPKM values. Coding gene and pseudogene annotations are from GENCODE project (v19). Values in parentheses show the average gene expression changes of the two replicates. A-B, C-D, E-F, G-H show the results for the SE or PE mapping for human K562 cells, liver, lung and ovary tissue, respectively.
(JPG)
