# Supplementary material for: AlignerBoost: A Generalized Software Toolkit for Boosting Next-Gen Sequencing Mapping Accuracy Using a Bayesian-Based Mapping Quality Framework
Source: PLoS Comput Biol. 2016 Oct 5;12(10):e1005096. doi: 10.1371/journal.pcbi.1005096 (PMC5051939; doi:10.1371/journal.pcbi.1005096)
Supplement: S1 Table — (DOCX) [file pcbi.1005096.s001.docx]

**S1 Table. Running options used for testing the default output of datasets.**

| Aligner name | SE datasets | | PE datasets | |
| --- | --- | --- | --- | --- |
|  | Non-default options | Notes | Non-default options | Notes |
| Bowtie | -q --sam --best  --phred33-quals | "--best" for enabling best mode | -q --sam --best  --phred33-quals  --minins <int>  --maxins <int> | "--minins/--maxins" for limiting insert size |
| Bowtie2 | -q --phred33 | Reporting best hit by default | -q --phred33  --minins <int>  --maxins <int> | "--minins/--maxins" for limiting insert size |
| BWA-MEM |  | best hit by default |  | best hit by default |
| SeqAlTo |  | best hit by default |  | best hit by default |
| Tophat2 | --solexa-quals  --no-sort-bam | best hit by default | --solexa-quals  --no-sort-bam | best hit by default |
| STAR | --outStd SAM | best hit by default | --solexa-quals  --no-sort-bam | best hit by default |
| Stampy | --maxbasequal 120 | To allow base quality higher than 50 | --maxbasequal 120 | To allow base quality higher than 50 |
| BatAlign |  | best hit by default |  | best hit by default |
