# Supplementary material for: AlignerBoost: A Generalized Software Toolkit for Boosting Next-Gen Sequencing Mapping Accuracy Using a Bayesian-Based Mapping Quality Framework
Source: PLoS Comput Biol. 2016 Oct 5;12(10):e1005096. doi: 10.1371/journal.pcbi.1005096 (PMC5051939; doi:10.1371/journal.pcbi.1005096)
Supplement: S2 Table — SD: standard deviation. (DOCX) [file pcbi.1005096.s002.docx]

**S2 Table. Parameters for generating simulated NGS datasets. SD: standard deviation.**

| Name | Source | Mean size | SD. size | Min size | Max size | Read length |
| --- | --- | --- | --- | --- | --- | --- |
| Genome | Non-gap genomic region^1^ | 400 | 80 | 150 | 650 | 100 |
| refExome | RefSeq exons | 250 | 50 | 100 | 400 | 100 |
| Pseudogene | VegaPseudogenes | 250 | 50 | 100 | 400 | 100 |
| RMSK | Repeat-Masker repetitive regions^2^ | 300 | 60 | 100 | 500 | 100 |
| refGene | RefSeq mRNAs | 150 | 30 | 75 | 225 | 75 |

All genomic regions are from UCSC hg19 assembly.

^1^Gaps are consecutive "N"s of the size of 10,000 that were padded for unassembled genomic gaps

^2^Only transposable elements annotated by RMSK are included; tandem repeats, simple repeats, low complexity and RNA repeats are excluded.
