# Supplementary material for: AlignerBoost: A Generalized Software Toolkit for Boosting Next-Gen Sequencing Mapping Accuracy Using a Bayesian-Based Mapping Quality Framework
Source: PLoS Comput Biol. 2016 Oct 5;12(10):e1005096. doi: 10.1371/journal.pcbi.1005096 (PMC5051939; doi:10.1371/journal.pcbi.1005096)
Supplement: S3 Table — AlignerBoost: AlignerBoost filtered best hits; Default: “default” best hits. (DOCX) [file pcbi.1005096.s003.docx]

**S3 Table.** Mapping sensitivity and precision of simulated DNA-seq single-end (SE) datasets by picking “best” hits with or without applying AlignerBoost procedures. AlignerBoost: AlignerBoost filtered best hits; Default: “default” best hits.

| Dataset | Aligner | AlignerBoost | | | Default | | |
| --- | --- | --- | --- | --- | --- | --- | --- |
|  |  | Precision | Sensitivity | F1 score | Precision | Sensitivity | F1 score |
| Genome | SeqAlto | 99.64% | 95.52% | 0.9754 | 97.50% | 96.79% | 0.9715 |
|  | Bowtie | 98.79% | 85.25% | 0.9152 | 96.42% | 82.25% | 0.8877 |
|  | Bowtie2 | 98.55% | 95.44% | 0.9697 | 96.28% | 96.14% | 0.9621 |
|  | BWA | 99.59% | 94.11% | 0.9677 | 97.21% | 97.21% | 0.9721 |
| RefExome | SeqAlto | 99.91% | 96.21% | 0.9803 | 98.17% | 97.59% | 0.9788 |
|  | Bowtie | 99.80% | 86.13% | 0.9246 | 97.66% | 83.18% | 0.8984 |
|  | Bowtie2 | 99.68% | 96.71% | 0.9817 | 97.73% | 97.69% | 0.9771 |
|  | BWA | 99.90% | 94.67% | 0.9721 | 97.84% | 97.84% | 0.9784 |
| Pseudogene | SeqAlto | 99.05% | 80.21% | 0.8864 | 88.52% | 86.64% | 0.8757 |
|  | Bowtie | 98.28% | 71.37% | 0.8269 | 84.38% | 71.94% | 0.7766 |
|  | Bowtie2 | 97.66% | 80.16% | 0.8805 | 86.71% | 86.58% | 0.8664 |
|  | BWA | 98.95% | 79.08% | 0.8791 | 87.54% | 87.54% | 0.8754 |
| RMSK | SeqAlto | 99.09% | 93.24% | 0.9608 | 95.58% | 94.52% | 0.9505 |
|  | Bowtie | 96.73% | 82.67% | 0.8915 | 93.42% | 79.83% | 0.8609 |
|  | Bowtie2 | 95.75% | 91.43% | 0.9354 | 92.08% | 91.90% | 0.9199 |
|  | BWA | 98.96% | 92.10% | 0.9541 | 95.37% | 95.37% | 0.9537 |
