# Supplementary material for: AlignerBoost: A Generalized Software Toolkit for Boosting Next-Gen Sequencing Mapping Accuracy Using a Bayesian-Based Mapping Quality Framework
Source: PLoS Comput Biol. 2016 Oct 5;12(10):e1005096. doi: 10.1371/journal.pcbi.1005096 (PMC5051939; doi:10.1371/journal.pcbi.1005096)
Supplement: S4 Table — AlignerBoost: AlignerBoost filtered best hits; Default: “default” best hits. (1) NA values are for NGS aligners that don’t support reporting all alignments under PE mode, thus AlignerBoost filtering was ineffective. (DOCX) [file pcbi.1005096.s004.docx]

**S4 Table.** Mapping sensitivity and precision of simulated DNA-seq paired-end (PE) datasets by picking “best” hits with or without applying AlignerBoost procedures. AlignerBoost: AlignerBoost filtered best hits; Default: “default” best hits. ⑴ NA values are for NGS aligners that don’t support reporting all alignments under paired-end mode, thus AlignerBoost filtering was ineffective.

| Dataset | Aligner | AlignerBoost | | | Default | | |
| --- | --- | --- | --- | --- | --- | --- | --- |
|  |  | Precision | Sensitivity | F1 score | Precision | Sensitivity | F1 score |
| Genome | SeqAlto | NA ⑴ | NA ⑴ | NA ⑴ | 98.62% | 98.61% | 0.9862 |
|  | Bowtie | 99.31% | 77.10% | 0.8681 | 97.87% | 67.51% | 0.7990 |
|  | Bowtie2 | 99.49% | 97.65% | 0.9856 | 98.33% | 98.29% | 0.9831 |
|  | BWA | NA ⑴ | NA ⑴ | NA ⑴ | 98.58% | 98.58% | 0.9858 |
| RefExome | SeqAlto | NA ⑴ | NA ⑴ | NA ⑴ | 98.49% | 98.49% | 0.9849 |
|  | Bowtie | 99.96% | 77.04% | 0.8701 | 98.07% | 70.11% | 0.8176 |
|  | Bowtie2 | 99.88% | 97.64% | 0.9874 | 98.43% | 98.41% | 0.9842 |
|  | BWA | NA ⑴ | NA ⑴ | NA ⑴ | 98.34% | 98.34% | 0.9834 |
| Pseudogene | SeqAlto | NA ⑴ | NA ⑴ | NA ⑴ | 92.07% | 92.07% | 0.9207 |
|  | Bowtie | 98.90% | 68.39% | 0.8086 | 87.28% | 60.43% | 0.7142 |
|  | Bowtie2 | 98.63% | 86.96% | 0.9243 | 91.71% | 91.66% | 0.9168 |
|  | BWA | NA ⑴ | NA ⑴ | NA ⑴ | 92.04% | 92.04% | 0.9204 |
| RMSK | SeqAlto | NA ⑴ | NA ⑴ | NA ⑴ | 97.93% | 97.92% | 0.9792 |
|  | Bowtie | 97.77% | 75.78% | 0.8538 | 96.21% | 61.65% | 0.7514 |
|  | Bowtie2 | 97.58% | 95.27% | 0.9641 | 95.68% | 95.50% | 0.9559 |
|  | BWA | NA ⑴ | NA ⑴ | NA ⑴ | 97.97% | 97.97% | 0.9797 |
