# Supplementary material for: AlignerBoost: A Generalized Software Toolkit for Boosting Next-Gen Sequencing Mapping Accuracy Using a Bayesian-Based Mapping Quality Framework
Source: PLoS Comput Biol. 2016 Oct 5;12(10):e1005096. doi: 10.1371/journal.pcbi.1005096 (PMC5051939; doi:10.1371/journal.pcbi.1005096)
Supplement: S5 Table — (1) DNA-seq aligner without local alignment ability, so 1DP function of AlingerBoost was enabled; (2) DNA-seq aligners; (3) RNA-seq aligners. (DOCX) [file pcbi.1005096.s005.docx]

**S5 Table.** Mapping sensitivity and precision of simulated RNA-seq single-end (SE) datasets by picking “best” hits with or without applying AlignerBoost procedures. ⑴ DNA-seq aligner without local alignment ability, so 1DP function of AlingerBoost was enabled; ⑵ DNA-seq aligners; ⑶ RNA-seq aligners.

| Dataset | Aligner | AlignerBoost | | | Default | | |
| --- | --- | --- | --- | --- | --- | --- | --- |
|  |  | Precision | Sensitivity | F1 score | Precision | Sensitivity | F1 score |
| refGene | Bowtie ⑴ | 98.27% | 85.98% | 0.9171 | 96.99% | 75.32% | 0.8479 |
|  | Bowtie2 ⑵ | 97.92% | 95.85% | 0.9687 | 96.49% | 85.83% | 0.9085 |
|  | BWA ⑵ | 97.43% | 94.30% | 0.9584 | 96.88% | 96.86% | 0.9687 |
|  | Tophat2 ⑶ | 99.50% | 94.82% | 0.9710 | 97.88% | 94.46% | 0.9614 |
|  | STAR ⑶ | 99.64% | 95.19% | 0.9736 | 97.57% | 96.20% | 0.9688 |
