# Supplementary material for: AlignerBoost: A Generalized Software Toolkit for Boosting Next-Gen Sequencing Mapping Accuracy Using a Bayesian-Based Mapping Quality Framework
Source: PLoS Comput Biol. 2016 Oct 5;12(10):e1005096. doi: 10.1371/journal.pcbi.1005096 (PMC5051939; doi:10.1371/journal.pcbi.1005096)
Supplement: S6 Table — (1) DNA-seq aligner without local alignment ability, so 1DP function of AlingerBoost was enabled; (2) DNA-seq aligners; (3) RNA-seq aligners; (4) NA values are for NGS aligners that don’t support reporting all alignments under PE mode, thus AlignerBoost filtering was ineffective. (DOCX) [file pcbi.1005096.s006.docx]

**S6 Table.** Mapping sensitivity and precision of simulated RNA-seq paired-end (PE) datasets by picking “best” hits with or without applying AlignerBoost procedures. ⑴ DNA-seq aligner without local alignment ability, so 1DP function of AlingerBoost was enabled; ⑵ DNA-seq aligners; ⑶ RNA-seq aligners; ⑷ Aligners that don’t support reporting multiple-mapped PE-reads, thus filtering was ineffective.

| Dataset | Aligner | AlignerBoost | | | Default | | |
| --- | --- | --- | --- | --- | --- | --- | --- |
|  |  | Precision | Sensitivity | F1 score | Precision | Sensitivity | F1 score |
| refGene | Bowtie ⑴ | 98.62% | 77.66% | 0.8689 | 97.05% | 61.61% | 0.7537 |
|  | Bowtie2 ⑵ | 98.84% | 95.93% | 0.9736 | 96.71% | 86.13% | 0.9111 |
|  | BWA ⑵ | NA ⑷ | NA ⑷ | NA ⑷ | 97.23% | 97.22% | 0.9723 |
|  | Tophat2 ⑶ | 99.55% | 94.94% | 0.9719 | 98.15% | 94.71% | 0.9640 |
|  | STAR ⑶ | 99.88% | 96.14% | 0.9797 | 98.29% | 97.68% | 0.9798 |
