# Supplementary material for: AlignerBoost: A Generalized Software Toolkit for Boosting Next-Gen Sequencing Mapping Accuracy Using a Bayesian-Based Mapping Quality Framework
Source: PLoS Comput Biol. 2016 Oct 5;12(10):e1005096. doi: 10.1371/journal.pcbi.1005096 (PMC5051939; doi:10.1371/journal.pcbi.1005096)
Supplement: S7 Table — Both HapMap phase3 (HapMap3) and 1000genomes (1000G) datasets were downloaded from GATK FTP bundles. All statistics are based on best hits without mapQ cutoff. (DOCX) [file pcbi.1005096.s007.docx]

**S7 Table.** **Comparison of mapping precision and sensitivity of real exome-seq datasets by incorporating known SNP information for AlignerBoost.** Both HapMap phase3 (HapMap3) and 1000genomes (1000G) datasets were downloaded from GATK FTP bundles. All statistics are based on best hits without mapQ cutoff.

| Target enrichment method | Type | Mapping precision | | | Average coverage depth (X) | | |
| --- | --- | --- | --- | --- | --- | --- | --- |
|  |  | AlignerBoost filtered | AB + HapMap3 SNP | AB + 1000G SNP | AlignerBoost filtered | AB + HapMap3 SNP | AB + 1000G SNP |
| SureSelect | SE | 59.960% | 59.962% | 59.961% | 8.247 | 8.246 | 8.247 |
| Haloplex |  | 98.330% | 98.331% | 98.330% | 8.028 | 8.028 | 8.028 |
| SeqCap |  | 70.846% | 70.845% | 70.848% | 6.917 | 6.916 | 6.917 |
| TruSeq |  | 77.418% | 77.418% | 77.418% | 10.165 | 10.166 | 10.165 |
| SureSelect | PE | 58.412% | 58.414% | 58.413% | 16.489 | 16.489 | 16.489 |
| Haloplex |  | 98.388% | 98.389% | 98.388% | 15.974 | 15.975 | 15.975 |
| SeqCap |  | 71.082% | 71.084% | 71.085% | 14.312 | 14.311 | 14.313 |
| TruSeq |  | 76.934% | 76.935% | 76.935% | 20.149 | 20.151 | 20.151 |
