# Supplementary material for: AlignerBoost: A Generalized Software Toolkit for Boosting Next-Gen Sequencing Mapping Accuracy Using a Bayesian-Based Mapping Quality Framework
Source: PLoS Comput Biol. 2016 Oct 5;12(10):e1005096. doi: 10.1371/journal.pcbi.1005096 (PMC5051939; doi:10.1371/journal.pcbi.1005096)
Supplement: S8 Table — Note that AlignerBoost supports filtering customized SAM/BAM alignment files produced by ANY NGS aligners. However, for the aligners listed below, AlignerBoost supports automatic generation of executable scripts with fine tuned options aiming to boost both the precision and sensitivity of the alignments. BWT: Burrows–Wheeler transform algorithm. (DOCX) [file pcbi.1005096.s008.docx]

**S8 Table.** **NGS aligners currently supported by AlignerBoost**. Note that AlignerBoost supports filtering customized SAM/BAM alignment files produced by ANY NGS aligners. However, for the aligners listed below, AlignerBoost supports automatic generation of executable scripts with fine-tuned options aiming to boost both the precision and sensitivity of the alignments. BWT: Burrows–Wheeler transform algorithm

| Aligner name | Type | 1DP enabled by default? | Notes |
| --- | --- | --- | --- |
| Bowtie | BWT based, DNA-seq | Yes | No Smith-Waterman local alignment algorithm support |
| Bowtie2 | BWT based, DNA-seq | No |  |
| BWA/BWA-MEM | BWT based, DNA-seq | No | Preferred algorithm of the authors |
| BWA-SW | BWT based, DNA-seq | No | Recommended for long reads |
| BWA-ALN | BWT based, DNA-seq | Yes | Original version for short reads |
| NovoAlign | Hash based, DNA-seq | No | Only non-commercial version tested |
| SeqAlto | Hash based, DNA-seq | No | MD:Z mismatch score of the latest version is ill-formatted; need to enable –fix-MD option |
| TopHat1 | RNA-seq | No | TopHat with Bowtie engine |
| TopHat2 | RNA-seq | No | TopHat with Bowtie2 engine |
| STAR | RNA-seq | No | Requires large RAM |
