# Supplementary figures and images for: AlignerBoost: A Generalized Software Toolkit for Boosting Next-Gen Sequencing Mapping Accuracy Using a Bayesian-Based Mapping Quality Framework
Source: PLoS Comput Biol. 2016 Oct 5;12(10):e1005096. doi: 10.1371/journal.pcbi.1005096 (PMC5051939; doi:10.1371/journal.pcbi.1005096)

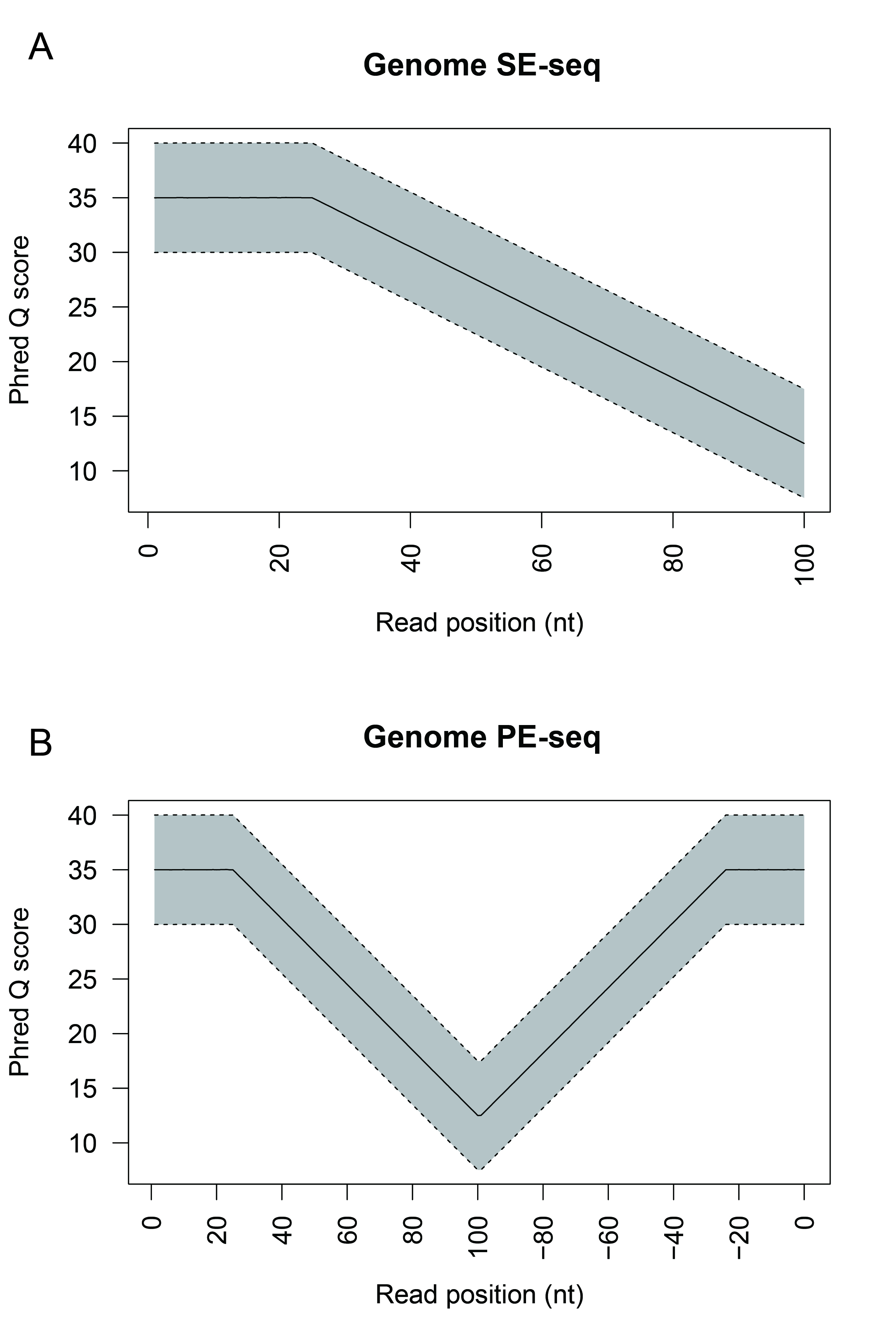

Supplement: S1 Fig — Y-axes indicate the read quality scores in Phred-scale; X-axes indicate the base-pair positions with positive and negative values representing forward and reverse read positions, respectively; Center solid lines: mean values of the quality scores at corresponding positions; shaded areas between dashed lines: mean ± SD (standard deviation) of the quality scores at corresponding positions; all quality scores were drawn from truncated Gaussian distributions. A: Quality score distributions for the simulated SE-dataset; B: Quality score distributions for the simulated PE-dataset. (JPG) [file pcbi.1005096.s009.jpg]

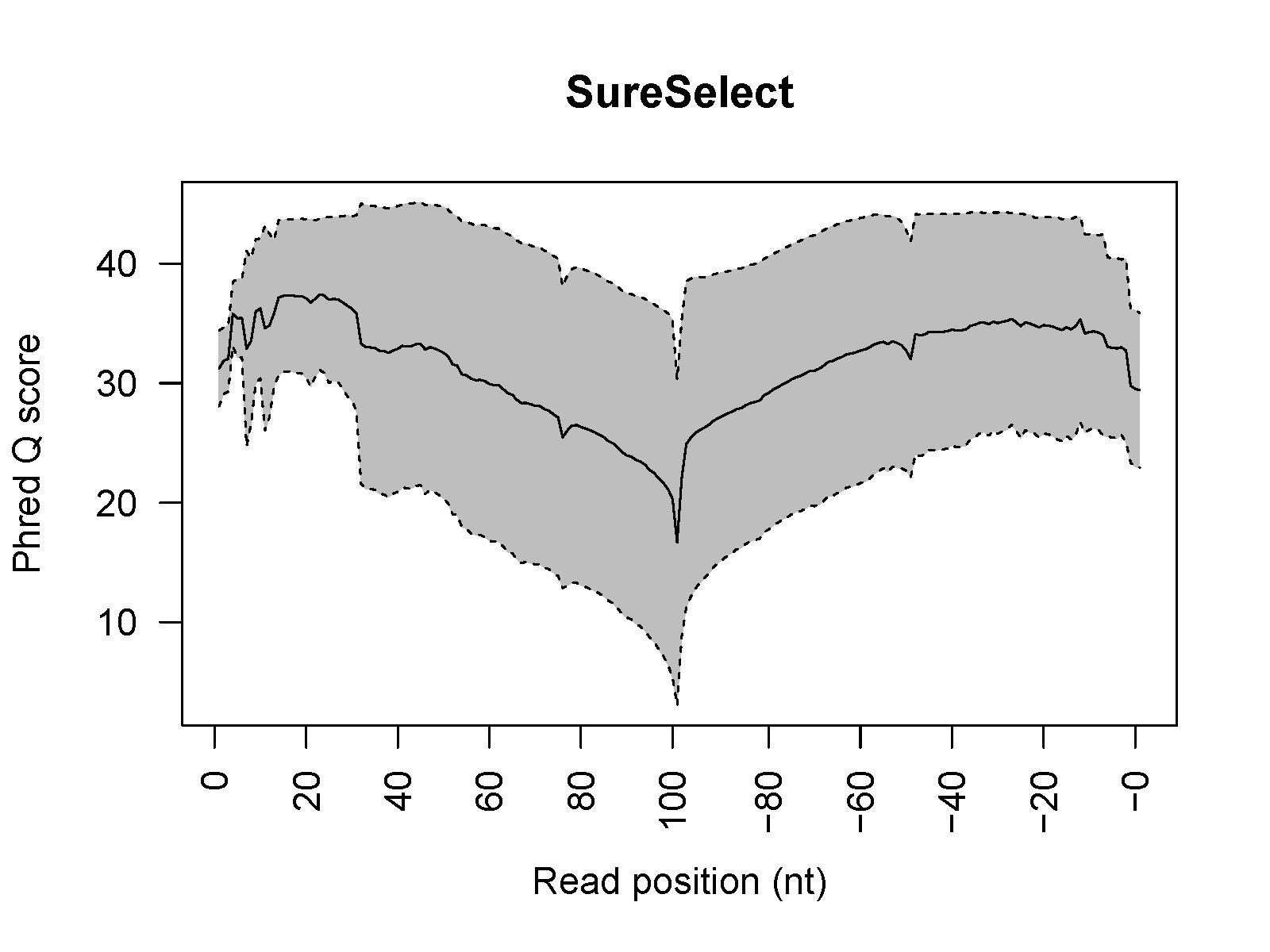

Supplement: S2 Fig — Y-axes indicate the read quality scores in Phred-scale; X-axes indicate the base-pair positions with positive and negative values representing forward and reverse read positions, respectively; Center solid lines: mean values of the quality scores at corresponding positions; shaded areas between dashed lines: mean ± SD (standard deviation) of the quality scores at corresponding positions. (JPG) [file pcbi.1005096.s010.jpg]

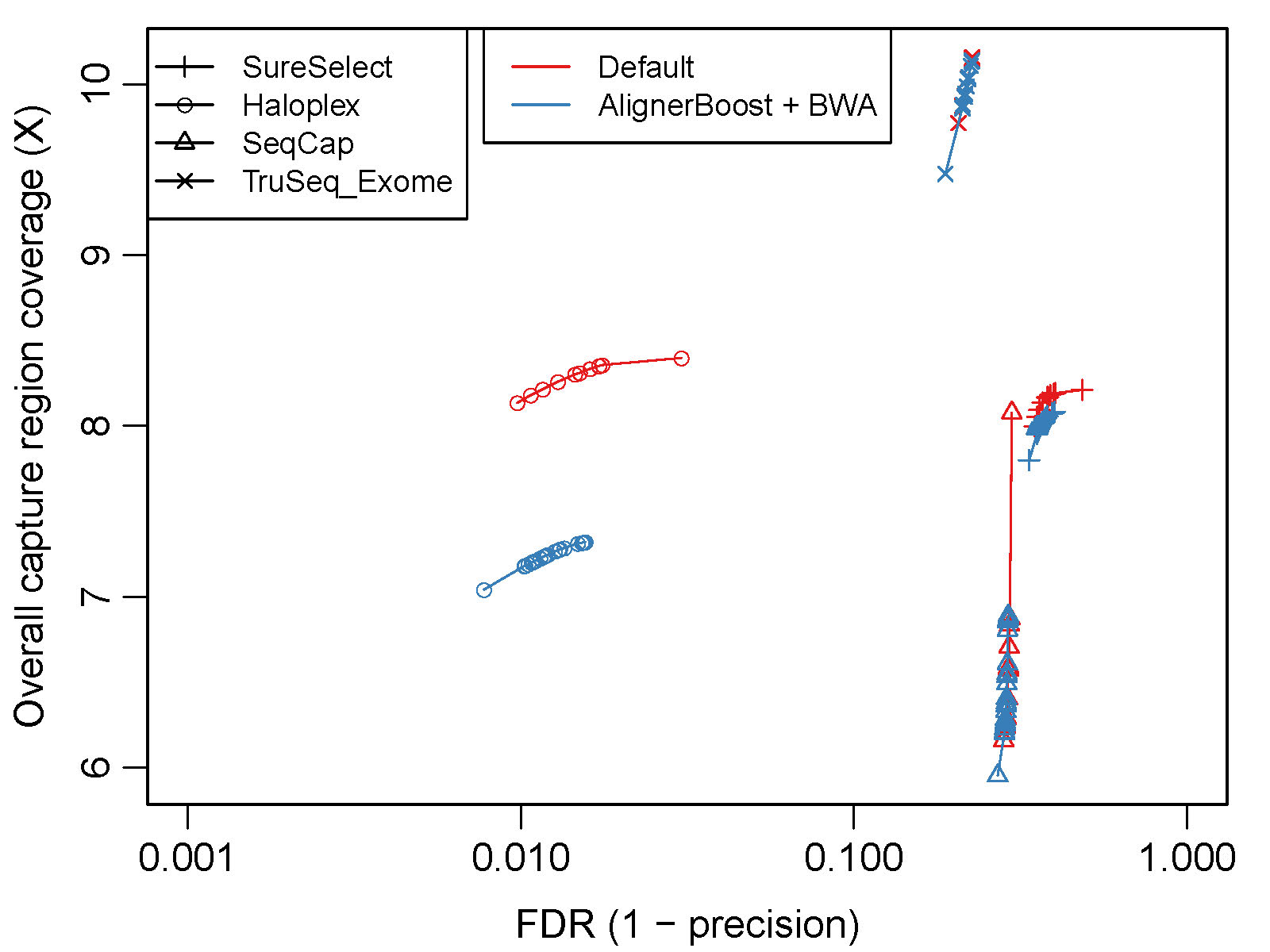

Supplement: S3 Fig — All mappings were performed using BWA-MEM (BWA). The mapQ varies from 0, 3, 6, 10, 13, 20, then in increments of 10 up to the maximum allowed values of the indicated aligner. “Default” indicates aligners’ default best hits; “AlignerBoost” indicates best hits via AlignerBoost mapping and filtering procedures. (JPG) [file pcbi.1005096.s011.jpg]

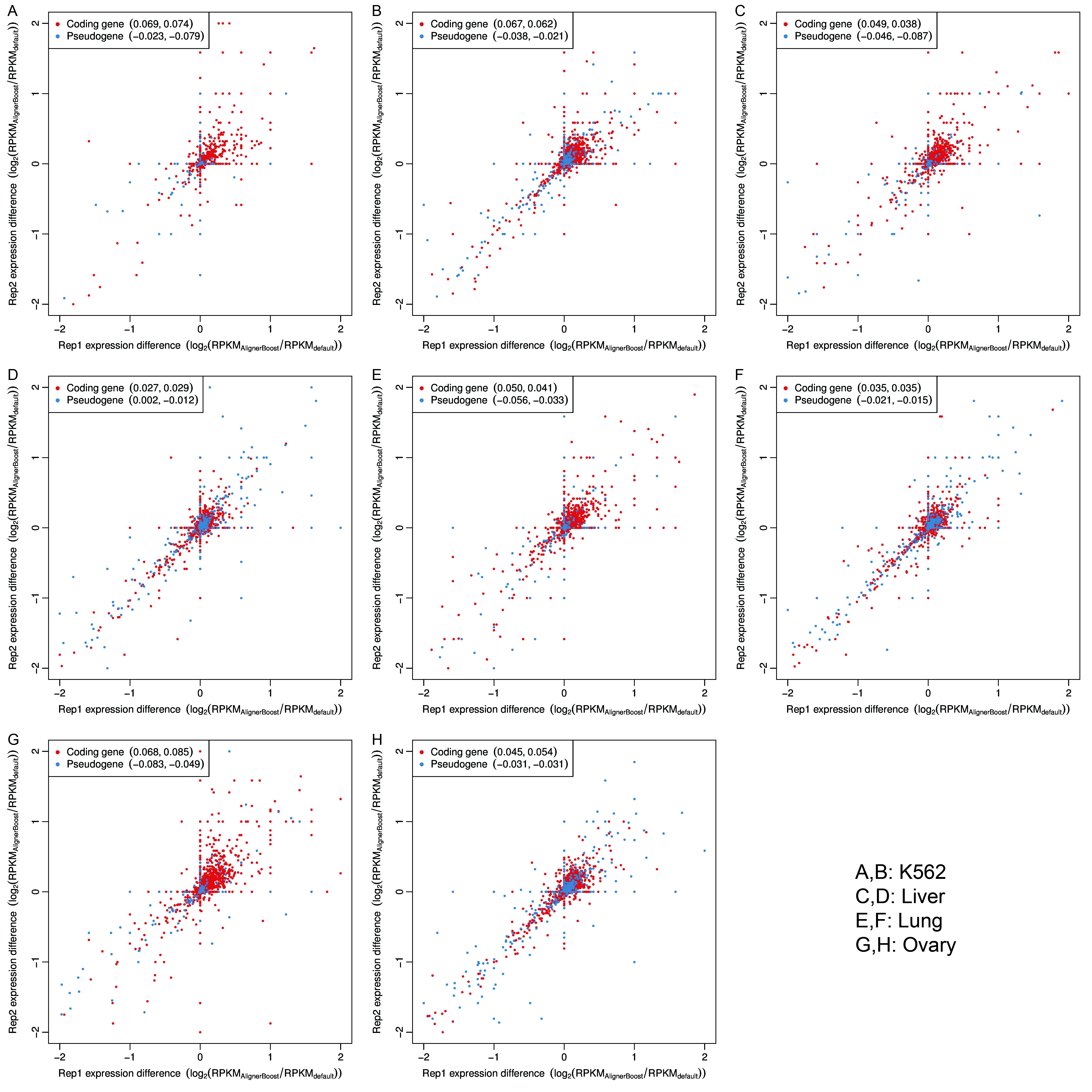

Supplement: S4 Fig — Gene expression represented by RPKM values. Coding gene and pseudogene annotations are from GENCODE project (v19). Values in parentheses show the average gene expression changes of the two replicates. A-B, C-D, E-F, G-H show the results for the SE or PE mapping for human K562 cells, liver, lung and ovary tissue, respectively. (JPG) [file pcbi.1005096.s012.jpg]
